# Telehealth interventions for alcohol use disorder: A systematic review^☆^

**DOI:** 10.1016/j.livres.2022.08.004

**Published:** 2022-08-31

**Authors:** Arpad Kelemen, Elizabeth Minarcik, Christopher Steets, Yulan Liang

**Affiliations:** a Department of Organizational Systems and Adult Health, University of Maryland, Baltimore, MD, USA; b Department of Family and Community Health, University of Maryland, Baltimore, MD, USA

**Keywords:** Alcohol use disorder (AUD), Liver disease, Cognitive behavioral therapy (CBT), Telehealth, Digitally delivered, Systematic review

## Abstract

Alcohol use disorder (AUD) is a worldwide problem for individuals of varying ages and backgrounds and is associated with the underlying cause of alcoholic liver disease, liver cirrhosis, liver cancer, or many other common diseases and health conditions. Existing treatments such as cognitive behavioral therapy (CBT) have been demonstrated as an evidence-based treatment to aid individuals struggling with AUD. However, these treatments have excessive costs and time demand with trained experts. In this paper, we examine the efficacy and long-term impacts of digitally delivered CBT and other online telehealth tools and apps for AUD patients. Results show the effectiveness in the ability of digitally delivered CBT to decrease alcohol use in AUD patients. The additional use of online technologies and smartphone apps for post-CBT care demonstrates that such computer-aided apps could have long-term effects when it is continually employed, which opens the door for AUD patients who were not seeking treatment elsewhere. Further longitudinal examination research is needed to evaluate the lasting effects in liver health and other chronic diseases associated with digitally delivered alcohol reduction for AUD patients.

## 1 Introduction

### 1.1 Background

Alcohol use disorders (AUD) are among the most damaging, costly common disease conditions globally, afflicting many individuals with severe physical, mental, and social problems.^1^ The estimated one-year and lifetime prevalence of AUD are 13.9% and 29.1%, respectively, with half of who with lifetime AUD having a severe disorder. In 2019, approximately 14.5 million Americans aged 12 and older had AUD and an estimated 414,000 adolescents (aged 12 to 17) had AUD.^2^^,^^3^ Abusive alcohol consumption causes around 95,000 deaths every year, making alcohol the third leading preventable cause of death in the United States (US). In addition, alcohol-impaired driving fatalities accounted for 31% of overall driving fatalities. Lastly, hazardous or excessive alcohol use increases the risk of developing chronic diseases and other serious alcohol-related health problems over time, including liver disease/cirrhosis, cardiovascular disease, stroke, cancers, high blood pressure, and other digestive conditions.^4^

AUD has known contributions to over 200 diseases and adverse health outcomes.^5^ From liver disease alone, in 2018, of the 83,517 liver disease deaths among individuals aged 12 and older, 47.8% involved alcohol.^6^ In 2019, of the 85,688 liver disease deaths among individuals aged 12 and older, 43.1% involved alcohol.^7^^,^^8^ Among all liver cirrhosis deaths, 47.9% were alcohol-related in 2013, which increased to 49.5% in 2015. The proportion of alcohol-related cirrhosis deaths was the highest (76.8%) among persons aged 25 to 34, followed by persons aged 35 to 44, at 72.7% in 2015.^9^^,^^10^ Excessive alcohol consumption is the second most common indication for liver transplantation, accounting for approximately 40% of all primary liver transplants in Europe and about 25% in the US.^11^ From 2010 to 2016, alcohol-related liver disease was the primary cause of almost one in three liver transplants in the US, including the total number of transplants necessitated by alcoholic cirrhosis, alcoholic liver disease and hepatitis C virus infection, and 40% of transplants necessitated by hepatocellular carcinoma. Alcoholic chronic liver disease has replaced hepatitis C virus infection as the leading cause of liver transplantation.^12^ Unfortunately, of the individuals with AUD who received a formal diagnosis, fewer than four million patients received any type of treatment.^13^ Among those aged 12 and older who had AUD in 2018, only 7.2% received any treatment.^7^

### 1.2 Existing AUD diagnosis and therapies

An AUD diagnosis and at-risk alcohol consumption can be identified with the Alcohol Use Disorders Identification Test (AUDIT). AUDIT is a ten-question screening tool developed by the World Health Organization to recognize hazardous alcohol consumption. AUDIT scores of 8–15 indicate dangerous and reckless alcohol use, while scores over 16 denote alcohol dependence.^14^

The most common evidence-based AUD treatment is cognitive behavioral therapy (CBT), which has conventionally been provided by a clinician to an individual patient or group.^15^ The focus of CBT is to identify the core problem and learn ways to combat the problem through stress reduction, coping techniques, and goal setting.^16^ Treatment of AUD with CBT requires specially trained professionals and numerous hours of face-to-face consultations. The second common treatment for AUD is Alcoholics Anonymous (AA), which features a 12-step program that functions as a free social support network to guide recovery.^2^

CBT and AA's 12-step program have many similarities. Both treatments address the cognitive and physical problems that AUD patients experience and promote the need to interact socially in a different manner than when they were using alcohol. Both treatments depend on social interaction and involve multiple participants. The idea is that coping and problem-solving skills, as well as social support groups will help maintain patient sobriety and decrease the chance of relapse.

The major difference between these two is that CBT focuses on cognitive restructuring of thought and behavioral processes and is an evidence-based practice performed primarily by clinicians, while AA identifies itself as “a spiritual program of action,” wherein it does not rely on any specific religious sect but acknowledges a power greater than oneself. Currently, there is little education given in proper medical institutions that go over the 12-step AA program in comprehensive depth. For that reason, most clinicians use CBT in clinical settings. While many studies have been done around AA meetings, they do not normally happen in clinical settings, other than occasionally renting a room in a medical facility.^2^

Both CBT and AA therapies have shown potential success in treating AUD when followed. However, of the millions of patients diagnosed with AUD, only 10% actively seek such treatments.^17^ The low percentage follow through with receiving AUD treatments is due to the stigma associated with AUD, the excessive cost of treatment, and poor access to care. This highlights the need to determine more flexible easy access solutions that provide more treatment options.^2^

### 1.3 Roles of computer-aided online technology for AUD treatments

As smartphone and app technology become popular and more available, especially during the COVID-19 pandemic, so has the capacity for individual patients to seek and find online treatments. For example, multiple websites about AUD and the beginning steps to address it can be found through www.alcoholcheckup.com, www.downyourdrink.org.uk, and www.soberistas.com.^18^, ^19^, ^20^ Each of these websites offers tools to evaluate a patient's personal knowledge of their alcohol abuse, their willingness to seek therapy, and after providing limited personal information, can help postulate which therapy may be the most beneficial. For instance, the Soberistas website offers the most comprehensive treatment assistance out of the above three websites. It uses five core features for AUD therapy, including personal stories; subjected blogs and forums; the ability to ask individual questions to a doctor; webinars that give expert advice; and chat rooms that offer support and discussion in real time. These are the first steps towards gaining control and finding the correct treatment plan. Despite these available resources, telehealth with online treatment is still a new medium for AUD patients.

Computer online technology and digitally delivered interventions may play several roles in AUD treatments that help overcome some of the obstacles preventing patients from seeking classic therapies. For instance, it offers a more informal setting (such as using Zoom) instead of the in-person settings traditionally used in CBT and AA. Using a selected computer technology may help individuals recognize that their level of alcohol use is no longer recreational and therefore needs change. So, they may better understand their situation and seek help. Also, online therapies allow for more anonymity.^18^ Individuals do not need to show up to an AA program meeting where they might be recognized, and they do not need to receive a formal diagnosis from a clinician. Online, patients may also not feel the shame of telling a person intimate and often embarrassing details from their life. When a patient either cannot attend or does not wish to attend therapies with person-to-person contact, computer-aided AUD therapies can offer an alternative as a new treatment setting to limit the embarrassment associated with treatments.^18^

Moreover, computer-aided AUD therapies could reduce the prohibitive costs needed for CBT and bypass common hurdles of transportation, childcare, or inability to meet a clinician's specific schedule.^17^ It can treat a diverse AUD population and reduce or eventually eliminate the need for trained specialists. An additional key role of computer online technology is as a post-acute AUD therapy preventative tool. For example, the Alcohol-Comprehensive Health Enhancement Support System (A-CHESS) is a new program to assess long-term effects and outcomes for patients' ability to avoid relapse after the therapies end.^13^ These areas and roles where telehealth with computer online technology and AUD therapy may interact form the basis and highlight the importance of this review.

## 2 Materials and methods

The research articles to evaluate the effectiveness of digitally delivered computer online technology-based CBT for AUD therapy were collected utilizing the PubMed and Cumulative Index for Nursing Allied Health Literature (CINAHL) databases. Two separate independent searches and screening relating to the above discussed two AUD therapies (CBT and AA program) were conducted. The first search used the keywords “cognitive behavioral therapy”, “AUD”, and “alcohol abuse” to generate a list of related articles. The resulting articles were further narrowed by year – 2013 to 2020 – and type of article – peer-reviewed, randomized controlled trial, systematic review, or Meta-analysis. This yielded 429 articles for further review. The articles' titles were screened for relevance to AUD and CBT interventions delivered digitally. Ten studies were selected for further review; five articles were excluded for involving substance abuse problems other than alcohol or mental problems. Four randomized controlled trials (RCT) and one Meta-analysis were chosen to assess the effectiveness of digitally delivered CBT on reducing alcohol consumption.

The second search focuses on evaluating the long-term impact of other telehealth tools and smartphone technology for AUD; therefore, the search was set for articles published between 2000 and 2020 using the keywords “Alcoholics Anonymous”, “alcoholism”, “telehealth”, “online”, and “smartphone”. The search with keywords “alcoholism” and “online” yielded 5292 articles; with “alcoholism” and “telehealth” just yielded 442 articles; and with the keywords “alcoholism” and “smartphone” yielded 309 articles. These articles were further narrowed down to five articles to highlight the changes of telehealth and computer technology makes to AUD treatments, which include two cross-sectional studies, two qualitative studies, and one RCT. In addition, one article was used to understand some of the framework and differences between CBT and the AA 12-step program for AUD. This article was selected based on its significance in the history and current therapies in addition to its connection to this review. Overall, ten studies resulted from two independent searches were evaluated to examine the utility of digitally delivered computer technology for AUD patients.

### 2.1 Digitally delivered CBT interventions for AUD

The first five articles included the effectiveness of digitally delivered CBT, as compared to the traditional face to face CBT interventions to treat AUD and reduce the amount of alcohol consumed. Summaries of the four RCT and the one Meta-analysis are included below as well as in Table 1.^15^^,^^17^^,^^21^, ^22^, ^23^

**Table 1:** Evidence of digital delivered CBT treatments for AUD.

Author and reference	Evidence type	Objective	Sample, eligibility	Methods, intervention, comparison	Results, outcomes	Limitations
Kiluk *et al*.^15^	RCT	(i) To examine CBT delivered via a web-based format would be more effective than standard treatment in decreasing alcohol use and number of days of alcohol use. (ii) To study the effectiveness of a stand-alone web-based CBT.	Participants (*n* = 68) from an outpatient substance abuse facility in Connecticut. Eligibility: 18 years or older; able to read at a 6^th^ grade reading level; met the DSM-IV criteria for alcohol abuse, and free of an untreated mental disorder.	Participants were randomly assigned to one of three groups for CBT treatment over eight weeks. (i) TAU weekly group/individual meetings with certified counselors via the outpatient substance abuse facility. (ii) TAU plus CBT4CBT, the web-based CBT treatment containing 7 modules on CBT concepts. (iii) CBT4CBT with monitoring, consisting of short weekly meetings to check-in reviewing use of the web-based CBT treatment and answer questions. Self-reported assessment of alcohol use was taken weekly during treatment, and 1, 3 and 6 months following treatment end.	Participants with either CBT4CBT (63% and 65%) treatment were more likely to complete the whole course than those receiving only TAU (26%); (*P* < 0.01). Participants in all treatments reduced their alcohol consumption. TAU with CBT4CBT decreased alcohol use the quickest compared to other treatments. Those receiving more treatment had more favorable outcomes.	(i) Small sample size with poor retention. (ii) Many participants, especially those receiving TAU did not receive treatment due to not showing up to meetings.
Zill *et al*.^21^	RCT	To test the effectiveness of a CBT internet-based intervention called Vorvida compared to CAU for AUD.	Participants (*n* = 608) were from multiple sources including insurance companies, primary care physicians' offices, and through the media. Eligibility: 18 years or older; had internet access, had an AUDIT score over three, and no impairment in hearing, speaking, or vision.	Participants were randomized by a computer into two groups. (i) Vorvida: an internet-based intervention using a broca software system. The system was organized into 4 modules for different CBT techniques including goal setting, self-monitoring, handling cravings and risk management. The program included the participant's weekly assessment of alcohol consumption and a mood check. Participants were given access for 6 months. (ii) CAU did not receive Vorvida until after the study. Participants were free to seek outside help if desired. Participants' alcohol use was assessed at baseline, 3, and 6 months using self-reported 7-day and 30-day recall.	Vorvida group significantly decreased more alcohol consumption over the last 30 days and last 7 days as compared to the CAU group at both 3 months and 6 months (*P* < 0.001). Vorvida group decreased their alcohol consumption from baseline to six months by 31.02 g, compared to CAU who only dropped their consumption by 17.89 g. One hundred and eighty-three participants were drop out, 37% from Vorvida group and 23% from CAU group.	(i) Relied on self-reporting only for alcohol consumption. (ii) The sample consisted of mainly highly educated and employed individuals. (iii) No true control. (iv) High dropout rates. (v) No follow-up after the completion of the study. (vi) No face-to-face interaction with participants.
Sinadinovic *et al*.^22^	RCT	To compare the effectiveness of three interventions to treat AUD: an online personalized feedback intervention, eScreen.se; an online CBT intervention, Alkoholhjalpen.se; and a control group.	Participants (*n* = 633) were recruited online through links given to people searching alcohol-related problems. Eligibility: participants needed to have an AUDIT score ≥6 for women and ≥8 for men, no drug use, ability to use a computer, and be over the age of 15.	Participants were randomized by a computer into three groups. (i) The control group, no intervention. (ii) eScreen.se, provided feedback to participants based on 2 in-depth self-reporting assessments. The assessments generated personalized normative feedback and graphs noting participant's alcohol use compared to normal levels. The feedback reflected the answers given during the assessments. (iii) Alkoholhjalpen.se, provided CBT techniques to participants through 18 modules, including problem solving, goal setting, refusal skills, dealing with cravings, and relapse prevention. Assessment of participants' alcohol use was conducted at 3, 6, and 12 months.	Over half of the participants were women with a mean age of 44 and an AUDIT score denoting alcohol dependence. Participants significantly dropped their alcohol use at 3 months (*P* < 0.001). However, no further decreases in alcohol use occurred at 6 and 12 months. Alkoholhjalpen.se had the lowest alcohol use noted at 3 months and 6 months but was not maintained at 12 months.	(i) eScreen.se and Alkoholhjalpen.se are available to the public for free, interventions could have been used by control group or other interventions. (ii) Control group could have been influenced by being asked about alcohol consumption. (iii) High dropout noted.
Hester *et al*.^23^	RCT	To examine the effectiveness of SMART Recovery (SR) and Overcoming Addictions (OA) web-based applications.	Participants (*n* = 189) were recruited through SR meetings and website. Eligibility: consume ≥5 drinks for men and ≥4 drinks for women of alcohol daily, had an AUDIT score ≥8, were new to SR, had internet access, and a desire to abstain from drinking.	Participants were randomized by a computer into three interventions. (i) SR: an alcohol withdrawal program which focuses on motivation, handling urges, controlling feelings, and creating a balanced life based on CBT theories. Participants could attend meetings of their choices. (ii) OA: web-based application was created based on the SR and contained 5 modules. The application had videos, audio, passages, links and graphics. Emails and text messages were sent to remind participants to login. (iii) SR + OR, participants had access to both interventions. Participants' alcohol use was tracked at baseline and 3 months based on a 90-day recall.	Majority of participants (60%) were female and mean education level was college educated. All three intervention groups decreased their alcohol intake compared to baseline (*P* < 0.001). This indicated the effectiveness of all interventions in reducing alcohol consumption. But no statistical significance was noted between interventions.	(i) No true control was present in the study. (ii) Relied on self-reporting for alcohol use. (iii) Participants were well educated.
Kiluk *et al*.^17^	Meta-analysis	To examine the evidence for CBT delivered via technology (CBT Tech) for the treatment of AUD. Specifically, focused on CBT content being distributed through the computer or mobile device.	The search of PubMed, Cochrane Register, EBSCO and PsycINFO yielded 10,145 studies. Based on the inclusion criteria: between 1990 and 2019, RCT, 18 years or older, AUDIT score >8, and CBT intervention via a digital format. Ultimately, 15 studies were selected.	Studies were divided into 4 comparisons based on the interventions: (i) CBT Tech compared to minimum/no treatment. (ii) CBT Tech compared to TAU. (iii) CBT Tech + TAU compared to TAU. (iv) CBT Tech compared to CBT delivered via a trained specialist. Results were further divided into early and late follow-ups. Early was defined as 1–3 months and late was defined as 6–12 months. Effect sizes were calculated based on similar studies, rather than15 articles. The standardized mean difference was used as the effect size metric.	CBT Tech versus no treatment showed CBT Tech perform better at early follow-up (*g* = 0.20, *P* = 0.03), late follow up with no statistical significance (*g* = 0.20, *P* = 0.09). CBT Tech versus TAU showed similar results between both interventions at early follow-up (*g* = −0.33, *P* = 0.09), late follow-up (*g* = −0.10, *P* = 0.49). CBT Tech + TAU compared TAU demonstrated CBT Tech + TAU outperforming at both early (*g* = 0.30, *P* = 0.003) and late follow-up (*g* = 0.31, *P* = 0.02). CBT Tech versus CBT in person showed no difference in results (*g* = −0.22, *P* = 0.15). CBT tech is a suitable intervention.	(i) Analysis only used studies with the goal of reducing alcohol consumption instead of alcohol-related problems or improvements in functioning. (ii) Small number of articles to use. (iii) Effect sizes were impacted by dividing studies.

Abbreviations: AUD, alcohol use disorders; AUDIT, Alcohol Use Disorders Identification Test; CAU, care as usual; CBT, cognitive behavioral therapy; RCT, randomized controlled trials; SMART, Self-Management and Recovery Training; TAU, treatment as usual.

In the first study by Kiluk *et al.*,^15^ the researchers sought to determine if CBT delivered via a web-based format could outperform traditional face-to-face methods of CBT using an RCT over eight weeks. Sixty-eight participants were gathered from an outpatient substance abuse treatment center in New Haven, Connecticut. Participants' alcohol use was assessed weekly during the study, at the end of the study, and at 1, 3, and 6 months following the completion of the study. During these assessments, the researchers tracked the participants' amount of drinking with daily self-reports. Additionally, participants were evaluated for use of drugs. Urine samples were collected, and a breathalyzer was used to verify the participants' self-reporting.

The participants completed a pre-assessment noting their drinking habits for 4 weeks prior and were randomized by a computer into one of three treatment groups. The first treatment group (*n* = 22) received standard treatment as usual (TAU), which included weekly individual or group sessions with a certified counselor. Participants in this group were also allowed to use any available outpatient services at the treatment center. The second treatment group (*n* = 22) received TAU as well as the online CBT training (called CBT4CBT). CBT4CBT training consisted of seven modules focusing on distinct aspects of CBT treatment, such as drink refusal skills and coping with cravings. Each module took under an hour to complete and followed the counselor led training. Information was delivered via videos, interactive exercises, games, and homework assignments. The third group's (*n* = 24) treatment consisted of CBT4CBT plus monitoring. Participants had access to online training modules and met briefly with a counselor once a week to monitor the status of module completion and answer questions.^15^

Over half of the participants in the study were male (65%) and African American (54%), with an average age of 42. Only 52% completed the full treatment protocol of their designated group. Those in either group consisting of CBT4CBT were more likely to complete the treatment than the participants in TAU (TAU = 26%, TAU plus CBT4CBT = 65%, CBT4CBT plus monitoring = 63%; chi-squared *P* < 0.01). Participants in all three treatments reduced their alcohol consumption. Participants in TAU with CBT4CBT decreased alcohol use more quickly and had a greater number of days abstinent from drinking as compared to those in TAU only. Those individuals with access to the web-based format had higher retention rates and were more likely to complete the treatment. CBT4CBT together with TAU had the best long-term outcome for participants at six months. The participants in CBT4CBT plus TAU were more likely to have more treatment and therefore better outcomes. One limitation of this study is the poor retention rate in a moderate sample size.

In the second study completed by Zill *et al.*,^21^ researchers aimed to investigate the effectiveness of an internet-based intervention called Vorvida against care as usual (CAU) using an RCT. Vorvida used a Broca software system to interact with the participants in attempts to hold a dialogue. The system was organized into four modules, each teaching different CBT techniques including goal setting, self-monitoring, handling cravings, and risk management. A computer randomized the participants (*n* = 608) into two groups. One group (*n* = 306) received access to the Vorvida system for six months during the study. The other group (*n* = 302), CAU, would receive access to the Vorvida system following the completion of the study. Participants in the latter group could seek outside assistance if desired during the study. Alcohol consumption was tracked through two self-reporting tools, the Quantity-Frequency-Index (QFI) and the Timeline-Follow-Back (TFB), a 30-day recall and a 7-day recall, respectively. Data was collected using the two self-reporting tools at baseline, 3, and 6 months.

Results showed that the Vorvida group significantly reduced alcohol use at both 3 months and 6 months as compared to the CAU group (all *P* < 0.001). Participants in the Vorvida group decreased their alcohol consumption from baseline to six months by 31.02 g, as compared to the CAU group, who only dropped their consumption by 17.89 g. Additionally, those individuals in the Vorvida group noted fewer binge drinking days (4.6 days) within the last 30 days as compared to the CAU group (14.5 days). The limitations of this study included a high dropout rate, losing 183 participants, 37% from the Vorvida group and 23% from the CAU group. Other limitations included alcohol consumption measurements relying solely on self-reporting, no follow-up after the completion of the study, no true control was noted, but participants were mostly well educated with jobs.

The third RCT study (*n* = 633) completed by Sinadinovic *et al*.^22^ compared three interventions for the treatment of AUD. Assessments of a participant's alcohol use were conducted at 3, 6, and 12 months of self-reporting. The first group (*n* = 211) received access to an online feedback intervention website, https://eScreen.se/, which provided personalized normative intervention based on assessments completed by participants. These assessments generated feedback as well as graphs noting the alcohol use of the participants as compared to normal usage. The second treatment group (*n* = 212) received access to an online CBT intervention website, https://Alkoholhjalpen.se/, which offered CBT content across 18 modules. The modules comprised of exercises, passages for reading, and videos that cover a wide range of topics including risk assessment, decision making tools, goal setting, alcohol refusal training, dealing with cravings, and relapse deterrence. The website included content for reading, exercises, and videos. Participants also had the ability to document in a diary and communicate with other participants using the website. The third group is a control with no intervention (*n* = 210). Emails were sent to these participants in this group at the designated assessment times to self-report their alcohol consumption habits.

Over half of the participants were women (55.7%), had a mean age of 44 with an AUDIT score denoting alcohol dependence. The participants significantly dropped their alcohol use at 3 months as compared to baseline (*P* < 0.001). However, no further decreases in alcohol use occurred at 6 months and 12 months, only maintaining this level throughout the 12-month assessment. Alkoholhjalpen.se had the lowest alcohol use and was the most effective in reducing alcohol consumption noted at 3 months and 6 months as compared to the other intervention and control group. Participants who sought out services outside of the study had better outcomes than those who only used the interventions provided in the study.

A limitation of this study was that both websites eScreen.se and Alkoholhjalpen.se are available to the public for free. These could have been used by the control group. Additionally, the control group could have been influenced by being asked about alcohol consumption. Past research has indicated that simply asking individuals about their alcohol consumption can lead to lower alcohol usage. Like the first two studies, this study also suffered from a high attrition rate, and alcohol consumption measurements relied solely on self-reporting.

In the study completed by Hester *et al.*,^23^ researchers examined the effectiveness of SMART (Self-Management and Recovery Training) Recovery (SR) and Overcoming Addictions (OA) web-based applications to treat AUD using an RCT. Participants (*n* = 189) were randomized by a computer into three intervention groups. The first group (*n* = 87) used only the SR program, which is an alcohol withdrawal program available throughout the US. The program focuses on motivation, handling urges, controlling feelings, and creating a balanced life based on CBT theories. The second interventional group (*n* = 19) only used the OA application which was created based on SR. The application contained five modules including videos, audio, passages, links, and graphics. In the final group (*n* = 83), SR plus OR, participants had access to both interventions and could use either intervention as often as they deemed fit. The participants' alcohol use was tracked at baseline and 3 months based on a 90-day recall.

The participants were mostly female (60%) and had a mean education level of a college education. All participants across three groups significantly decreased their mean drinks per day as compared to the baseline from 8.0 to 4.6 (*P* < 0.001). Participants from three groups also significantly increased their percent days abstinent from 44% to 72% (*P* < 0.001), which indicated the effectiveness of 3 interventions in reducing alcohol consumption. No statistical significance was noted between interventions. The limitations of this study included no control group, reliance on self-reporting of alcohol use, and the participants were well educated.

In the Meta-analysis completed by Kiluk *et al.*,^17^ researchers examined the evidence for CBT delivered via technology (CBT Tech) for the treatment of AUD. Specifically, the Meta-analysis focused on CBT content being distributed through the computer or mobile device. Fifteen studies were used to complete the Meta-analysis, including 10 studies recruited online, 3 from clinics, and 2 from college campuses. Effect sizes were calculated based on similar studies, rather than the collection of fifteen articles. The standardized mean difference was used as the effect size metric. Studies were divided into four groups, (i) CBT Tech compared to minimum/no treatment; (ii) CBT Tech compared to TAU; (iii) CBT Tech plus TAU compared to TAU; and (iv) CBT Tech compared to CBT delivered via a trained specialist. Results were further divided into early (defined as 1–3 months) and late follow-ups (defined as 6–12 months).

The sample sizes of the collection of studies ranged from 42 to 7935 with a mean size of 656. The mean age of participants was 39, 46% were female, and most of the studies were based outside of the US. More than half of the studies (60%) included moderate alcohol users. Tech interventions were divided into modules, which ranged from 4 to 62 modules. The first comparison between CBT Tech and no or minimum treatment showed CBT performing better at early follow-up (*g* = 0.20, *P* = 0.03). Late follow-up for these studies showed no statistical significance (*g* = 0.20, *P* = 0.09). The second comparison between CBT Tech and TAU showed no statistical significance between the two interventions, early follow-up (*g* = −0.33, *P* = 0.09) and late follow-up (*g* = −0.10, *P* = 0.49). The third comparison between CBT Tech plus TAU and TAU demonstrated CBT Tech plus TAU outperforming at both early (*g* = 0.30, *P* = 0.003) and late (*g* = 0.31, *P* = 0.02) follow-up. The final comparison between CBT Tech and CBT delivered via a trained specialist showed no statistical significance in result (*g* = −0.22, *P* = 0.15).

Overall, the CBT delivered through technology is a suitable intervention for decreasing alcohol consumption whether as a stand-alone treatment or an add-on to existing treatment. Standardizing the delivery of the CBT content would improve outcomes. Studies reviewed showed a wide range of ways to distribute CBT to participants. The limitation of this Meta-analysis included division of studies into similar intervention types, and the only studies included were interested in decreasing alcohol consumption, rather than using studies which sought to reduce the consequences of AUD.^17^

### 2.2 Effectiveness of other online interventions

To evaluate the effectiveness of computer online technology to promote useful AUD treatments, we further examine the differences and the similarities in using CBT and other online interventions such as AA 12 steps program. Table 2 provides the details of five selected evidence-based studies (two cross-sectional studies, two qualitative studies, and one RCT) regarding the study design, setting, samples, variables, results, and recommendations.^13^^,^^18^, ^19^, ^20^^,^^24^

**Table 2:** AUD patients interacting with computer online technology for CBT.

Author and reference	Evidence type	Setting, sample, and sample size	Variables and outcome measures	Results and recommendations	Limitations
Lieberman^18^	Cross-sectional study	Setting: Online questionnaire evaluation tool on the website alcoholcheckup.com. Sample: 1297 users who took the guided assessment of alcohol use.	Variables: This study focused on patients understanding of alcohol use, family history, understanding what abuse entails. Outcome measures: The tool used AUDIT to measure the patient's recognition of a problem, and their understanding of how much their drinking is maladaptive.	Patients included 53% male, with age averaged 35 years. The majority were employed. The patients reported an average of 29.5 drinks per week. The average AUDIT score was 17.3. Recommendations: This study can continue to identify patient's characteristics that need help and, in the future, can measure what therapy interventions worked successfully.	Patient's understanding of computer technology, and their comfort with sharing sensitive information over it.
Khadjesari *et al.*^19^	Qualitative study with semi-structured interviews	Setting: In person and telephone recorded interviews from patients who accessed the Down Your Drink (DYD) website. Sample: 18 people were interviewed (10 women and 8 men). Average age was 43 years.	Variables: Patients from the website had to register for the DYD trial. Most participants believed their drinking was a problem. Most interviews were 1 h. Outcome measures: Interviewers collected data regarding the participants' ability to recognize alcohol problem, type of help wanted, and barriers to seeking help.	Results: The data collected helped interviewers understand the type of online experience the participants were looking for. Recommendation: The interviewers following up with the participants to see if their selected online help lead to successful therapy.	Served more as a guide to the participants and interviewers than a preconceived framework towards preselected types of therapy.
Sinclair *et al.*^20^	Cross-sectional study	Setting: Online survey at Soberistas website. Sample: Over 32,550 registered users of the Soberistas website, there were 3800 active users at the time of the survey, and 438 people completed the survey.	Variables: The survey was divided into four sections with fixed responses from questions in each section (demographics, membership to the website, usage of the website, and alcohol consumption). Outcome measures: Numerical data was analyzed, and free-text responses that were then categorized into table for analysis.	Among 438 users completed the survey. 94.0% were female, 97.0% were Caucasian, 73.4% were employed, and 53.5% had already tried other alcohol abuse therapies. Recommendations: It would be to further survey and follow up survey to understand progress and longevity of accomplished therapy without relapse.	(i) The unreliability of a unanimous survey, with regards to truthfulness, actual demographics, and an inability to include outliers from a fixed response list. (ii) Follow up question to patients who use more than one type of therapy, which offered more personal accomplishment.
Chambers *et al*.^24^	Qualitative study with in-depth interviews	Setting: Telephone interviews of UK-based users of the Soberitas website. Sample: 31 website users (25 women and 6 men).	Variables: Levels of engagement were measured through recorded interviews and compared with demographic information and current levels of alcohol use. Outcome measures: The level of engagement was defined as “Lurking” or just browsing via website, actively participating with the website, and leading therapies on the website. A fourth result were patients who reached an “authentic identity” and maintain continued sobriety after treatment.	Highlighted how patients interact with the website and evaluated the patient responses. Recommendation: Focused on how the use of a non-12-step online program has helped patients' recovery. Mapping of areas that offered the most assistance would help with future studies.	(i) Focused on qualitative study only, did not calculate totals of success or failure. (ii) Small sample size. (iii) Did not group the sample into levels of engagement. (iv) Possible truthfulness when conducting phone interviews for patients who only interact online.
McTavish *et al*.^13^	RCT	Setting: After care for alcohol dependence therapy from two treatment agencies' regions: Midwest and Northeast. Sample: 349 AUD were selected to either controlled group of standard aftercare from therapy, or experimental group received Alcohol-Comprehensive Health Enhancement Support System (A-CHESS).	Variable: Experiment group given a smartphone to use an app; compared with control group (the standard aftercare given after alcohol abuse treatment). Outcome measures: The use of the smartphone app was measured with amount of use, areas of use within the app, and the length of time the app was used after treatment. This data was compared to the continues use and success of the patient post treatment.	The experimental group used the A-CHESS app heavily and they were able to sustain that use over time. Recommendations: Continued study to find the pages in the app that are most helpful to longevity of use.	(i) Limited to three categories of self-determination theory. (ii) The pages viewed count can be different from exposure, for instance, a 45-min podcast and a one paragraph summary are both measured equally.

Abbreviations: AUD, alcohol use disorders; AUDIT, Alcohol Use Disorders Identification Test; CBT, cognitive behavioral therapy; RCT, randomized controlled trials.

The first cross-sectional study includes 1297 participants that used an online evaluation tool AUDIT located at www.alcoholcheckup.com.^18^ This evaluation focuses on the Stages of Change Readiness and Treatment Eagerness Scale (SOCRATES), which measures the factors of the recognition of a problem, and the ambivalence of their drinking as maladaptive. This online evaluation tool is not a treatment but could indicate if the participants would be willing to further interact and start the process of future online AUD treatments.

The second qualitative study conducted by Khadjesari *et al*.^19^ focuses on the AUD patients' experience in searching for help online prior to active treatment, which acts as a prescreening tool and a precursor to joining a treatment program. This study follows a total of 18 people who were interviewed after using the assessment tool on the website www.downyourdrink.org.uk. Similar factors to the previous Lieberman study were evaluated, such as the problem recognition, the type of help wanted, and barriers to formal help seeking, including stigma and if they wanted to stop or slow down their drinking.

The cross-sectional study conducted by Sinclair *et al*.^20^ evaluated a treatment and support system for AUD patients using the Soberistas social networking website discussed earlier (www.soberistas.com). This website offers 12-step plans that anyone can fit to their own style of treatment through the myriad of services that the site offers. It enacts CBT interventions and SMART to understand alcohol problems as well as treatments. The study evaluated how the website works, the demographics using it, previous treatments, level of change sustained, length of sobriety and the degree of success found with online treatment. Out of 3800 active users, 438 completed the survey. Results show that most of the surveyed patients were female, lived with their children, and had never tried any other type of AUD support. Eighteen percent of the women reported over one year of abstinence from alcohol.

The qualitative study conducted by Chambers *et al.*^24^ also utilized the Soberistas website and performed 31 in-depth telephone interviews. Each participant was questioned about their level of use, whether they passively use the website, whether they are creating an identity through use of the website, or they are leading the way for others on the website. Results showed that this website offers successful treatments and with continued use can help maintain long-term sobriety. With more involvement with the website, more success can be achieved in preventing relapse. One limitation is that some of these online tools involve fees. For example, the Soberistas website has two levels of participation. One level requires a fee and grants the member access to the entire site and all the resources available on the site. The other level allows the individual to see the full site but does not give access to all the features.

The last RCT study focused on after care for patients following completion of a treatment program.^13^ The study included 349 patients who recently completed residential treatment for AUD from either a Midwest facility or a Northeast facility. The participants were randomly assigned into the standard after care program or the experimental group. The experimental group was given a smartphone with the A-CHESS app on the phone. Results showed that experimental group participants used the A-CHESS app heavily and continued to use the app over an extended period. For instance, in the first week, 94% were using the app, and at about 3 and a half months post treatment, 80% were still using the smartphone app. The results also indicated that, given the opportunity and application, graduates of an AUD treatment would continually use computer-based technology to maintain their sobriety. It demonstrated that the A-CHESS app on smartphones can provide easy access to support systems to prevent relapse and can act as a helpful tool to maintain sobriety and patient relapse. With smartphones and online apps being so prevalent and the convenience of always having such support only a few clicks away, computer online technology support systems have shown to be beneficial in long term rehabilitation.

## 3 Discussion and conclusion

Excessive alcohol use affects millions of Americans and the world's population; however, most individuals with AUD rarely choose to seek treatment. This is often associated with the stigma attached to alcohol treatment programs and limited access to treatment in addition to the prohibitive cost and require highly trained specialists. To combat these treatments' shortcomings, digitally delivered CBT interventions and other online telehealth tools and apps can be utilized to decrease alcohol use for AUD patients. However, the efficacy must be demonstrated to ensure proper treatment of AUD patients.

After evaluating the articles included in this systematic review, we conclude that digitally delivered CBT interventions would be beneficial for those who have either not found success with current treatment options or have avoided them due to the stigma or feelings associated with them or any other reasons. Digital delivery of CBT interventions is as effective as standard face to face CBT treatment for AUD patients. These interventions can reduce alcohol consumption at the same rate as conventional treatments and increase the viability of dispersing CBT to diverse populations. However, they do not provide superior treatment to patients with AUD as originally hypothesized. Digital CBT interventions can be used as stand-alone treatments as well as in addition to standard treatments for lasting alcohol reduction in AUD patients.

Through computer aided online tools, smartphone technology and digital media can reduce related expenses, save time, help AUD patients to begin the recovery process, and lessen the need for experts. Understanding the need for help and actively searching for help are factors for successful AUD therapies. The use of AUDIT, SOCRATES, and other evaluation tools to assess and screen for AUD are the first stage to receiving help through online tools that are available at any time, so the patient can use them at their discretion.

The reviewed studies demonstrated that alcoholics could use online screening tools to recognize and understand alcoholism and the problems associated with it. These online screening tools could be further utilized for evaluating what type of treatments would work best for each individual patient. After using the evaluation tools, the website Soberistas offers multiple core measures to create an individualized 12-step treatment plan. The success of the 12-step Soberistas online program shows that computer-based therapies can be beneficial and an important adjuvant therapy option. The included studies also show that continued use of this website shows evidence of successful lasting treatment for post care.

After the treatment plan is complete, there are additional support systems that can be used online to prevent relapse. This further demonstrates that telehealth with computer technology, at all stages of AUD therapy, is an efficacious and accessible intervention. All these studies posit that online therapies are not only useful but also correlate with success and sobriety maintenance. Using computer technology to bring new therapies to patients through online websites can open more doors for patients to receive AUD care.

Telehealth including web-based CBT and smartphone app use for AUD intervention were already on the rise before the COVID-19 pandemic and made use of these technologies and delivery options even more accepted and prevalent during this pandemic. Websites are generally easier to read and navigate on computers with large screens than on smartphones and may be the choice of patients at home. On the other hand, smartphone apps provide portability, accessibility, and habit to users who are on the road or at work often. Individual users likely develop preferences if they use websites on computers or smartphone apps. The current dominant trend is mobile app development. The reviewed articles mainly focused on the comparisons between the web-based CBT or smartphone apps with traditional standard interventions. Additional studies could be conducted to compare the effectiveness of AUD intervention of telehealth between the websites and smartphone apps, and other rising telehealth tools such as virtue reality technologies.

Implications for practice based on the reviewed studies suggest that the evidence supports advantages from integrating computer online technology into AUD therapies. The great disparity between individuals diagnosed with AUD and those seeking treatment emphasizes the need for additional interventions. By increasing the simplicity of screening tools, therapies, and relapse prevention tools, more alcoholics will find it easier and more accessible to get treatment. The changes in access to treatments from clinical or meeting settings towards universal availability whether at home, work, or on the road has the potential to encourage people not only to seek treatment, but also to continue receiving it. Patients who have social anxiety are negatively affected by the stigma of seeking help in public, or do not have the time and resources to attend a face-to-face therapy session but can use these recent technologies from start to finish. By including pre-screening, treatment, and after-care maintenance supports with computer online technology could significantly prevent relapse and help to maintain sobriety.

## Authors' contributions

A. Kelemen and Y. Liang contributed equally to this work, wrote the manuscript, interpreted analysis findings and provided critical revisions. E. Minarcik and C. Steets provided two independent article searches, contributed to drafting the tables and wrote the manuscript.

## Declaration of competing interest

The authors declare that they have no conflict of interest.

## References

[1] Rehm J, Shield KD. (2019). Global burden of alcohol use disorders and alcohol liver disease. Biomedicines.

[2] Breuninger MM, Grosso JA, Hunter W, Dolan SI. (2020). Treatment of alcohol use disorder: integration of Alcoholics Anonymous and cognitive behavioral therapy. Training Edu Professional Psychol.

[3] Centers for Disease Control and Prevention Excessive alcohol use. http://wonder.cdc.gov/mcd.html.

[4] National Cancer Institute (2018). Alcohol consumption. http://www.progressreport.cancer.gov/prevention/alcohol.%20Number%20of%20deaths%20from%20multiple%20cause%20of%20death%20public-use%20data%20file.

[5] Agyapong VIO, Juhás M, Mrklas K (2018). Randomized controlled pilot trial of supportive text messaging for alcohol use disorder patients. J Subst Abuse Treat.

[6] National Survey on Drug Use and Health (NSDUH) https://www.samhsa.gov/data/data-we-collect/nsduh-national-survey-drug-use-and-health.

[7] Center for Behavioral Health Statistics and Quality (2019).

[8] National Center for Health Statistics Mortality Data on CDC WONDER https://wonder.cdc.gov/mcd.html.

[9] Alcohol-Related Disease Impact (ARDI) Application. http://nccd.cdc.gov/DPH_ARDI.

[10] Singal AK, Guturu P, Hmoud B, Kuo YF, Salameh H, Wiesner RH (2013). Evolving frequency and outcomes of liver transplantation based on etiology of liver disease. Transplantation.

[11] Stickel F, Datz C, Hampe J, Bataller R. (2017). Erratum: pathophysiology and management of alcoholic liver disease: update 2016. Gut Liver.

[12] Yoon YH, Chen CM. (2016).

[13] McTavish FM, Chih MY, Shah D, Gustafson DH. (2012). How patients recovering from alcoholism use a smartphone intervention. J Dual Diagn.

[14] Babor TF, Higgins-Biddle JC, Saunders JB, Monterio MG (2001). https://www.who.int/publications/i/item/audit-the-alcohol-use-disorders-identification-test-guidelines-for-use-in-primary-health-care.

[15] Kiluk BD, Devore KA, Buck MB (2016). Randomized trial of computerized cognitive behavioral therapy for alcohol use disorders: efficacy as a virtual stand-alone and treatment add-on compared with standard outpatient treatment. Alcohol Clin Exp Res.

[16] Mayo Clinic (2019). Cognitive behavioral therapy. https://www.mayoclinic.org/tests-procedures/cognitive-behavioral-therapy/about/pac-20384610.

[17] Kiluk BD, Ray LA, Walthers J, Bernstein M, Tonigan JS, Magill M. (2019). Technology-delivered cognitive-behavioral interventions for alcohol use: a meta-analysis. Alcohol Clin Exp Res.

[18] Lieberman DZ (2005). Clinical characteristics of individuals using an online alcohol evaluation program. Am J Addict.

[19] Khadjesari Z, Stevenson F, Godfrey C, Murray E. (2015). Negotiating the ‘grey area between normal social drinking and being a smelly tramp’: a qualitative study of people searching for help online to reduce their drinking. Health Expect.

[20] Sinclair JM, E Chambers S, C Manson C. (2017). Internet support for dealing with problematic alcohol use: a survey of the soberistas online community. Alcohol Alcohol.

[21] Zill JM, Christalle E, Meyer B, Härter M, Dirmaier J (2019). The effectiveness of an internet intervention aimed at reducing alcohol consumption in adults. Dtsch Arztebl Int.

[22] Sinadinovic K, Wennberg P, Johansson M, Berman AH (2014). Targeting individuals with problematic alcohol use via web-based cognitive-behavioral self-help modules, personalized screening feedback or assessment only: a randomized controlled trial. Eur Addict Res.

[23] Hester RK, Lenberg KL, Campbell W, Delaney HD (2013). Overcoming addictions, a web-based application, and SMART recovery, an online and in-person mutual help group for problem drinkers, part 1: three-month outcomes of a randomized controlled trial. J Med Internet Res.

[24] Chambers SE, Canvin K, Baldwin DS, Sinclair JMA (2017). Identity in recovery from problematic alcohol use: a qualitative study of online mutual aid. Drug Alcohol Depend.

